# Influence of cultural beliefs and practices on unassisted homebirths in Afghanistan: A qualitative exploration

**DOI:** 10.1371/journal.pgph.0005870

**Published:** 2026-01-30

**Authors:** Ahmad Rashed Wassif, Maisam Najafizada, Shree Mulay

**Affiliations:** 1 Discipline of Pediatrics, Faculty of Medicine, Memorial University of Newfoundland, St. John’s, Newfoundland, Canada; 2 Division of Population Health and Applied Health Sciences, Faculty of Medicine, Memorial University of Newfoundland, St. John’s, Newfoundland, Canada; SUPA71 Co., Ltd, THAILAND

## Abstract

Unassisted homebirths persist in Afghanistan, accounting for approximately one-third of all births. Despite efforts to increase skilled birth attendance, unassisted homebirths remain high, especially in rural and conservative areas. This study explores the cultural beliefs and practices that influence unassisted homebirths, aiming to inform culturally sensitive skilled birth care. A qualitative case study approach was employed, focusing on Afghan refugees resettled in Canada. Data were collected between September 20 and October 3, 2022, through in-depth interviews with women who had experienced at least one unassisted homebirth in Afghanistan, as well as focus group discussions with separate groups of men and women. In these discussions, participants shared their experiences and observations of unassisted homebirths and skilled birth care facilities in their communities. The data were transcribed and then thematically analyzed using Atlas.ti. Findings revealed three main themes: positive, existential, and negative cultural influences. Positive influences, such as husbands’ education and women’s concerns about complications associated with unassisted homebirths, motivated some women to seek skilled care. Existential factors, including preparations for childbirth, seeking divine protection, the practices of *Dayas* (traditional birth attendants), and traditional diets and remedies, represented neutral cultural practices shaping homebirth experiences. Negative influences, such as perceptions of facility births as disgraceful, reliance on family elders’ decisions, and traditional norms favoring homebirths, significantly hindered access to skilled care. Unique cases, including secretive homebirths driven by traditions like women’s veiling and seclusion within families, as well as delays in dowries, underscore the sociocultural complexities surrounding unassisted homebirths. The findings underscore the need for culturally sensitive health interventions that respect traditional practices while promoting skilled birth attendance. Engaging elder family members in health initiatives, integrating dayas into the healthcare system, and incentivizing community midwives to provide home-based skilled care could help shift perceptions and improve maternal health outcomes in Afghanistan.

## Introduction

Evidence indicates that despite a growing trend toward hospital or medical facility-based deliveries in low-income countries, unassisted homebirths in those countries remain entrenched in cultural norms and traditions [1]. In Afghanistan, where cultural traditions wield a significant influence [2], approximately one-third of births still occur at home, typically without skilled attendants, often assisted by a *Daya* [traditional birth attendant] or family members [3]. In this study, unassisted homebirth refers to births occurring at home without skilled birth attendants, whether completely alone or assisted only by untrained relatives or dayas. This is concerning, given that global data shows homebirths are linked with higher maternal mortality rates [4,5].

The Ministry of Public Health of Afghanistan implemented a range of interventions aimed at promoting skilled birth attendance. These initiatives included community health education programs led by community health workers [CHWs] [6], the training and deployment of community midwives [7,8], and the provision of emergency obstetric and newborn care [EmONC] services through the Basic Package of Health Services [BPHS] and the Essential Package of Hospital Services [EPHS] [9]. However, after two decades of progress, the healthcare system faced a major setback following the government’s collapse in 2021, leading to catastrophic funding shortages and the withdrawal of many international donors and health organizations. Despite these challenges, key healthcare programs such as BPHS and EPHS continued to receive support from international donors, including the World Bank [10]. Research suggests that, for most people, the ability to reach healthcare facilities remained unaffected [11], and unassisted homebirths continue to be a common practice [12].

Afghanistan has a high fertility rate, with an average of 5.1 children per woman [13], and more than 80% of women do not use contraceptives [14]. This high fertility rate, coupled with cultural expectations, often compels women to undergo labour at home, particularly in remote areas [15]. Cultural norms dictate that women endure labour pains in silence, often delaying assistance until symptoms become severe [16,17], which can lead to complications [17]. Furthermore, some women feel ashamed to seek help from family members or healthcare providers for birth-related conditions [17,18]. In this study, cultural beliefs and practices refer to the shared perceptions, values, norms, and customary behaviours surrounding childbirth that are socially transmitted within Afghan families. Drawing on the PEN-3 cultural model, culture encompasses not only rituals or traditions but also the knowledge systems, household decision-making norms, gender expectations, and collective interpretations of childbirth risk that shape women’s experiences and choices [19,20].

Studies indicate that families in Afghanistan traditionally prefer [29] homebirths without skilled birth attendants, often assisted by dayas [21]. Home is perceived as a safe and comfortable place for birth, aligned with cultural identity and heritage [22,23]. Unassisted homebirths facilitated by family members or dayas are often preferred for their perceived alignment with cultural identity and heritage [22]. This reliance remains strong despite the increasing availability of skilled birth care, particularly in rural areas. National surveys have found that unassisted homebirths are more prevalent in rural families in Afghanistan [13,24], where people tend to be more traditional. Most people in Afghanistan live in extended families, which are often patriarchal in structure [25]. An extended family typically consists of multiple nuclear families linked by parent–child relationships, with an older man, his wife or wives, unmarried children, and married sons with their families living together or nearby [26]. In such families, decisions are generally made by the elders [25], which often include opting for a homebirth assisted by a daya due to her perceived expertise, affordability, and availability [27,28]. Some women or families also seek assistance from experienced older women in the community during childbirth [23]. Postpartum care following unassisted homebirths often involves traditional remedies or self-medication, particularly in rural areas where access to medical facilities is limited [17,18]. Traditional beliefs present a significant challenge, with some serious medical complications attributed to “evil spirits” [17,28]. In such cases, families often consult a *Mullah* (cleric), who is regarded as reliable, accessible, and cost-effective [18,22].

In addition to cultural influences, structural and systemic barriers—including political instability and security risks, difficult terrain and limited transportation infrastructure, severe health workforce shortages, and deteriorating health system capacity—also shape access to skilled birth care in Afghanistan [29,30]. This means that cultural beliefs and practices are shaped by broader social structures, including the availability, accessibility, and quality of maternity care. In settings where safe, respectful, and woman-centred services are limited, cultural norms may become more salient in guiding decisions about childbirth, including preferences for homebirth.

While existing literature provides valuable insights into homebirth practices in Afghanistan, it falls short of providing a comprehensive understanding of the cultural beliefs and practices that influence unassisted homebirths. This study aims to describe cultural beliefs and practices surrounding childbirth in Afghanistan and to identify those that influence women’s choices of birthplace and birth attendant. The intention is to examine how women themselves understood and expressed these cultural influences, while recognizing that structural barriers form part of the broader context in which these practices occur. By delving into the intricacies of culturally embedded childbirth traditions, this research aims to inform more effective and culturally sensitive birth care interventions. Ultimately, the goal is to improve maternal health outcomes by enhancing our understanding of homebirth practices in a traditional, collectivist society marked by its cultural heritage and unique challenges.

### Conceptual approach

**Fig 1:**
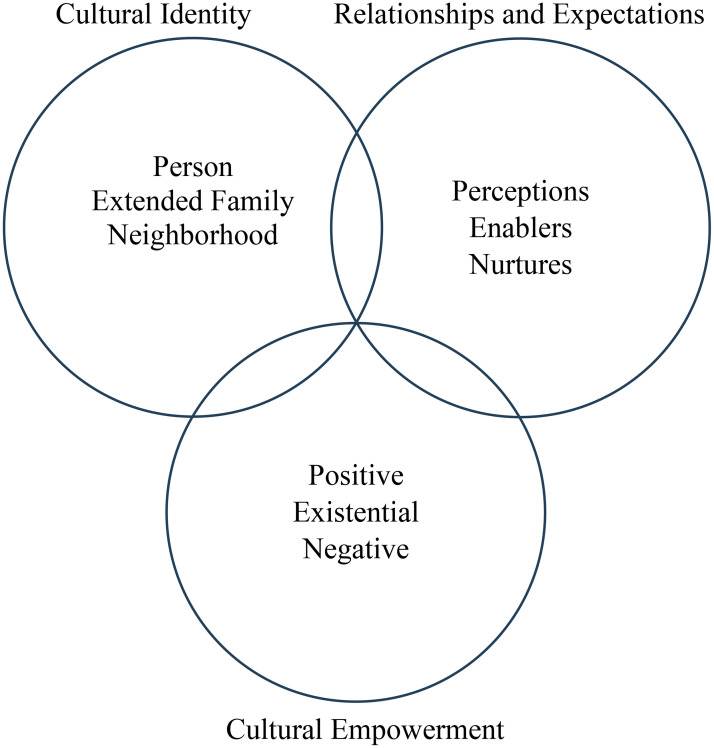
PEN-3 Cultural Model.

This study employs the PEN-3 cultural model to examine the influence of cultural factors on unassisted homebirths in Afghanistan. This model provides a suitable framework for explaining factors that have a positive, negative, or neutral influence on unassisted homebirths in Afghanistan. Introduced by Airhihenbuwa in 1989, the PEN-3 cultural model underscores the central role of culture in understanding health beliefs, behaviours, and outcomes, addressing its omission in earlier health behaviour theories [19,20]. The PEN-3 cultural model [Fig 1] is structured around three domains: Cultural Identity, which encompasses Person, Extended Family, and Neighborhood; Relationships and Expectations, which includes Perceptions, Enablers, and Nurturers; and Cultural Empowerment, which focuses on Positive, Existential, and Negative aspects [31].

The model has been applied primarily in two areas: assessing health problems and implementing health campaigns [31,32]. Most commonly, the assessment phase is used to explore community perspectives by integrating elements from the Relationships and Expectations and Cultural Empowerment domains into a three-by-three analytical framework [33].

This study adopts the assessment phase of the PEN-3 model, focusing specifically on the Relationships and

Expectations and Cultural Empowerment domains to investigate cultural influences on unassisted homebirths. The Cultural Identity domain, which centers on identifying intervention entry points [33,34], falls outside the scope of this study and is therefore not included.

Within the Relationships and Expectations domain, perceptions are defined as the knowledge, attitudes, values, and beliefs that influence health behaviors, either positively or negatively. Enablers refer to the social or institutional resources that affect health behaviors, while nurturers represent family and community traditions that shape these behaviors. The Cultural Empowerment domain examines three categories: positive factors that encourage engagement in health behaviors; existential factors, which reflect unique cultural characteristics that are neutral for health behaviors and should be incorporated into health interventions; and negative factors that discourage participation in health-promoting behaviors [31,35,36].

To avoid normative interpretation of the PEN-3 categories, it is important to clarify that the labels Positive, Existential, and Negative are not value judgments imposed by the researchers. Instead, they reflect how cultural practices function in relation to the health behaviour under study—in this case, women’s ability or decision to seek skilled childbirth care—and are grounded in the perspectives shared by participants. “Positive” refers to cultural influences that support health-enhancing behaviours; “Existential” refers to neutral cultural practices that are meaningful to the community and do not hinder care-seeking; and “Negative” refers to cultural expectations or norms that participants themselves described as limiting their ability to access skilled birth care. These categories serve as analytic tools within the PEN-3 framework, rather than as a hierarchy of cultural value. This study used a three-by three matrix [Table 3: PEN-3 Analytical Framework] that combines the Relationships and Expectations domain with the Cultural Empowerment domain.

**Table 3:** PEN-3 Analytical Framework.

Domain	Positive	Existential (neutral)	Negative
Perceptions	Concerns about birth complications in homebirths assisted by dayas	Pursuit of divine protection by offering prayers Belief about T*aweez* (amulet) from a mullah for smooth delivery	Perception of facility birth as *Sharm* [disgrace] Reliance on faith and destiny Facility delivery considered only for complications Trust in dayas as knowledgeable birth attendants
Enablers	Healthcare facilities visited for complications	Practices and advice of dayas in assisting homebirths	Dayas and relatives as birth attendants
Nurtures	Educated husbands in support of facility birth	Preparation of essentials for the mother and baby’s care Preparing the physical environment for childbirth Traditional dietary practices in homebirths Remedies and healing traditions in homebirths	Homebirth upheld as a traditional norm *(Rawaj)*. Deference to family elders’ decisions in childbirth Veiling and seclusion practices as barriers to facility-based childbirth Circumstances resulting from delayed *Toyana* or *Qaleen* (dowry) Childbirth in solitude

## Methods

This article is derived from the author’s doctoral thesis, which employed a single intrinsic case study design to explore unassisted homebirths in Afghanistan [37]. The content, including the methodological approach, findings, and interpretation, is adapted from that broader project for publication as a stand-alone article. The design of this study followed Stake’s inductive qualitative case study approach, which emphasizes studying a case within its real-life context and generating insights from participants’ perspectives [38]. The case under examination—unassisted homebirth—was defined within specific parameters: it was geographically situated in Afghanistan; contextually defined as unassisted homebirths examined as a social and health-related phenomenon influenced by cultural, gender, and healthcare system factors; and temporally framed between the post-2001 implementation of the BPHS and the EPHS [39,40] and the return of the Taliban regime in 2021. This timeframe was selected because it aligned with the post-2001 reconstruction of Afghanistan’s health system, during which substantial reforms and investments in maternal healthcare services were implemented.

This study initially aimed to purposively select participants from Nangarhar and Herat provinces in Afghanistan. However, the Taliban’s takeover of Afghanistan in August 2021 disrupted the planned data collection. The resettlement of thousands of Afghan refugees in Canada between 2021 and 2022 provided an opportunity to recruit participants with similar characteristics. Thus, Afghan refugees were invited through principal researcher's personal network of friends and community contacts, who shared the study invitation scripts widely within Afghan refugee communities in Toronto, reflecting a purposive sampling approach. Based on the information in the invitation scripts, interested individuals contacted the principal researcher, expressed their willingness to participate, and subsequently received a detailed briefing about the study over the phone. We invited those who had relocated to Canada within the 12 months prior to data collection.

Based on the inclusion criteria, in-depth interview [IDI] participants were women of childbearing age who had experienced at least one unassisted homebirth in Afghanistan within the ten years preceding data collection. This period was selected to capture a wide range of childbirth experiences while supporting meaningful recall and to align as closely as possible with the temporal boundary of the case study, which spans the period from 2001 to 2021 following the implementation of the BPHS and EPHS. Participants were invited to describe their own childbirth experiences and, where relevant, to share observations of childbirth practices among family members and relatives. Focus group discussions [FGDs] included Afghan refugee men and women who reflected on childbirth practices and the use of maternity care services in their communities across the full temporal span of the case study [2001–2021]. FGDs were designed to capture community-level norms, shared understandings, and perceived changes in childbirth practices over time. To maintain this broader focus, women participating in FGDs were not asked to discuss their personal childbirth experiences.

This approach ensured that participants were information-rich cases closely aligned with the aims of this study. This method is consistent with qualitative case study approaches described by Merriam, who emphasized the importance of choosing participants who can best inform the study through their knowledge and lived experiences [41]. Stake similarly supports purposive selection based on the richness of information each participant can provide in relation to the case [38]. Saturation was assessed throughout data collection; once successive IDIs and FGDs yielded no new themes, we determined that thematic saturation had been achieved. Although the number of IDIs in this study [n = 9] was modest, in relatively homogenous populations, conducting 6–7 IDIs generally uncovers the majority of the themes [42]. Similarly, Guest et al. indicate that two to four FGDs are typically adequate to capture major themes [43]. Therefore, information power and saturation are both used to guide sample adequacy in qualitative research.

We used semi-structured, open-ended questions in IDI and FGD guides, which were originally developed by the principal researcher in English following Billups’ protocols [44]. The IDI and FGD guides were translated into Dari and Pashto, then back-translated into English, following Brislin’s model of cross-cultural instrument translation to ensure conceptual equivalence [45,46]. Bilingual Afghan community members reviewed the translations to confirm cultural appropriateness. The guides were reviewed and approved by the principal researcher’s PhD thesis supervisory committee but were not pilot tested.

Data were collected between September 20 and October 3, 2022. We conducted IDIs with nine women and two FGDs: one with six men and the other with eight women. The IDIs were scheduled for approximately 60 minutes and the FGDs for 90 minutes. Women’s and men’s FGDs were conducted separately in order to respect cultural norms. Participants could speak in their preferred local languages [Dari or Pashto], and since both the principal researcher and the data collection assistant were bilingual and fluent in Dari and Pashto, IDIs and FGDs were conducted in the language most comfortable for participants. Only two IDIs were conducted in Pashto, as these participants preferred to speak in their native language. The remaining IDIs and both FGDs were conducted in Dari, which participants selected as their preferred language. The IDI process comprised three main phases: reconstructing unassisted homebirth experiences, providing detailed accounts by sharing narratives, and reflecting on overall experiences [47].

IDIs were facilitated by the principal researcher with the support of a trained female assistant, who initiated discussions to support culturally appropriate engagement. The principal researcher then used probing techniques to elicit detailed accounts of unassisted homebirths. To address potential recall bias, participants were encouraged to reconstruct their experiences chronologically, and follow-up probes were used to elicit contextual details, supporting depth and internal consistency across narratives.

FGDs followed a similar format, with the researcher leading discussions and the assistant contributing probes when necessary. Field notes were recorded in a research diary for contextual insights. No repeat IDIs or FGDs were required. This study received ethical approval from Memorial University’s Interdisciplinary Committee on Ethics in Human Research [ICEHR] in August 2022.

In this study, the findings from both IDIs and FGDs were analyzed and presented together. While IDI participants shared personal childbirth experiences that offered direct insights into the factors influencing their decisions to give birth at home, FGD participants contributed broader reflections based on their observations of family members, neighbors, and community norms in Afghanistan. Data from both sources were used in combination to develop a comprehensive understanding of how individual experiences and collective social contexts shape decisions around unassisted homebirths.

Audio recordings were transcribed and translated into English, with participant names replaced by codes to maintain anonymity. A thematic analysis approach was applied using Atlas.ti [ATLAS.ti 23, Scientific Software Development GmbH, Berlin, Germany]. Transcripts were read for familiarization and initial understanding of the data, after which relevant segments were inductively coded, without applying the PEN-3 categories. The PEN-3 framework was introduced only after themes had emerged, to organize the findings into its assessment domains. Following the coding of all transcripts, a textual coding report was generated. Codes were then reviewed, organized into overarching themes, and refined for coherence and distinctiveness, resulting in clearly defined themes and sub-themes. In this study, member checking with participants was not conducted due to logistical and contextual constraints.

## Results

### Participant demographics

A total of nine women [aged 29–42] who had experienced at least one unassisted homebirth were interviewed. Parity ranged from three to eight. Some participants primarily opted for unassisted homebirth; two delivered all their children at home without skilled attendants, whereas others reported between one to five unassisted homebirths. Two participants indicated that only their most recent birth was an unassisted homebirth. All homebirths occurred without skilled birth attendants, with most taking place among women residing in rural areas. The unassisted homebirths experienced by participants had occurred 3–9 years before the IDIs. As noted previously, seven out of nine IDI participants had experience of both unassisted homebirth and delivery in a hospital. Among these seven participants, three women had a single hospital delivery, two women had four hospital deliveries, and the remaining two had two and four hospital deliveries, respectively. See Table 1 for more details on the deliveries.

**Table 1:** Children Born to Participants and Their Place of Birth.

Participant	Number of children	Place of Birth			
		Urban		Rural	
		Health Facility	Home	Health Facility	Home
P1	3	2	1	0	0
P2	3	0	0	0	3
P3	4	0	0	1	3
P4	3	1	0	0	2
P5	6	3	0	1	2
P6	6	5	1	0	0
P7	5	4	1	0	0
P8	8	0	2	0	6
P9	6	5	1	0	0

Most of the IDI participants [seven out of nine] lived in extended families.

Three of the nine women interviewed had never attended school and were illiterate. Three participants had completed 10–14 years of education, likely representing the completion of secondary education, while three others had university-level education. Of all the IDI participants, only three were employed in Afghanistan and had a source of income, suggesting that the majority were primarily engaged in domestic duties rather than formal employment.

Eight women aged 31–48 and six men aged 38–55 participated in the FGDs. All participants in men’s and women’s FGDs had attained a university or similar level of education. With the exception of one female participant, who was a housewife, all were employed in either government or non-governmental organizations in Afghanistan.

Participants in this study, including those in both the IDIs and FGDs, were from various provinces of Afghanistan, including Kabul, Kandahar, Ghazni, Daikundi, Wardak, Parwan, Kapisa, Baghlan, Kunduz, Badakhshan, Nangarhar, and Paktiya. Table 2 provides the main characteristics of the participants.

**Table 2:** Characteristics of Participants.

Participants characteristics	IDI	FGD	
		Female	Male
Number	9	8	6
Age range	29-42	31-48	38-55
All children born at home	2	N/A	N/A
1-5 children born at home	7	N/A	N/A
No education	3	1	0
Education, 10–14 years	3	0	0
Education, University level	3	7	6
Unemployed	6	1	0
Employed	3	7	6

### Thematic findings

The findings of this study are organized and discussed using the PEN-3 cultural model analytical framework. Although the themes were developed inductively from the data, the matrix provided a culturally grounded structure for organizing the findings in the Results section. This approach made it possible to illustrate not only the types of cultural influences [perceptions, social resources, family traditions] but also how participants understood these influences as facilitating, neutral, or constraining in relation to decisions about childbirth location and care. The analysis identified three main themes: positive, existential, and negative cultural influences on unassisted homebirths. Drawing on the PEN-3 cultural model, culture includes not only rituals or traditions, but also knowledge systems, household decision-making norms, gender expectations, and collective interpretations of childbirth risk. Table 3 describes the analytical framework of the PEN-3 model adopted by this study.

### Theme 1: Positive cultural influences on unassisted homebirth

In this study, positive cultural factors or practices are those that encourage women to seek skilled birth attendants or avoid unassisted homebirths, encompassing positive perceptions, enablers, and nurturers.

### Positive perceptions

**Concerns about Birth Complications in Homebirths Assisted by Dayas:** Although these influences appeared at the personal or household level, participants consistently described them as shaped by widely shared community narratives about childbirth risk, indicating a culturally transmitted understanding of danger associated with unassisted homebirths that aligns with the PEN-3 definition of positive cultural perceptions supporting safer childbirth practices. The findings indicated that concerns about birth complications during homebirths assisted by dayas were a key factor influencing some women’s belief that unassisted homebirths are unsafe and prompting them to consider giving birth in a hospital instead. Participants shared stories of difficult homebirths, including babies who did not breathe at birth, retained placenta, heavy bleeding, and long-term complications such as infections and persistent bleeding. Furthermore, participants recounted instances from their relatives and communities in which the baby, the mother, or both died due to inadequate care at home. These participants believed that preventing such complications requires giving birth with a healthcare provider or in a healthcare facility. For example, participant 6 explained that she initially delivered at home but later recognized the dangers when she experienced severe bleeding after birth due to high blood pressure:

*I think delivery with a doctor is better. After delivering at home, I suddenly started bleeding because I had high blood pressure. IDI Participant #6.*

Similarly, participant 7 described long-term health consequences from homebirth without skilled care, which shaped her view that facility births are safer:

*I had bleeding for a long time after delivery at home. I had swelling, and I got microbes. Delivering at home without a doctor or midwife is dangerous. IDI Participant #7*

Participant 1 recalled the loss of her newborn and emphasized how the absence of skilled care during her first delivery led to complications and the death of her newborn:

*Giving birth at a health clinic is much better. After my first child was born at home, it did not cry. The child’s Jora (placenta) did not come out after the baby was born. When we took the baby to the hospital, its skin was pink. By the time we arrived at the hospital, the child had died. IDI Participant #1.*

### Positive enablers

**Healthcare Facilities Visited for Complications:** Visiting healthcare facilities for complications reflected a shared social norm rather than an individual decision. Participants described facility-based care as acceptable primarily when childbirth was perceived as risky or abnormal, a judgment typically made within families and communities. It was a culturally mediated pathway to skilled care, in which family approval, collective risk interpretation, and prior experiences shaped when and how women were permitted to seek medical assistance. These socially accepted pathways to care enabled contact with skilled providers and, for some women, influenced future views on the importance of skilled birth attendance. Accounts from two participants showed that women consulted available healthcare providers in their communities after facing complications during labour or when their previous childbirth experiences suggested the likelihood of complications. Furthermore, when home remedies proved ineffective in managing post-delivery complications, such as bleeding or high blood pressure, women sought medical care by visiting health facilities, doctors, or midwives. By engaging with formal healthcare services in response to complications, they accessed skilled care, received information on pregnancy and birth care, and recognized the importance of skilled attendance for all deliveries. Participant 6, who had experienced heavy bleeding after a homebirth, recalled how hospital treatment altered her perception of delivery with a doctor:

*I came to understand that delivery with a doctor in a hospital is important. After giving birth at home, I felt dizzy and lost a lot of blood. Then, I was taken to the hospital, where I was examined and given some medicine. Everyone in my family has high blood pressure. The doctor checked me and gave me sublingual pills. I was kept in the hospital until 2 p.m. and was given information about the importance of delivering in a clinic. My baby was also checked there. IDI Participant #6*

### Positive nurturers

**Educated Husbands in Support of Facility Birth:** Within Afghan extended families, husbands’ involvement in decision-making was culturally normative; thus, their education influenced not only individual choices but also household-level cultural expectations regarding safe childbirth. Participant accounts indicated that the husband’s education was a positive factor influencing decisions to deliver in a healthcare facility. Two participants shared stories of their educated brothers, who, living in nuclear families, encouraged their wives to deliver at healthcare facilities. This encouragement was often rooted in their awareness of the potential risks associated with unassisted homebirths and the importance of skilled birth attendance. In these cases, the absence of extended family dynamics, such as the influence of a mother-in-law, allowed the husbands to have a more direct role in their wives’ decisions to seek care at a healthcare facility.

*Those who are educated, like my brother, would take their wives to the doctor for delivery. IDI participant #7*

### Theme 2: Existential cultural influences on unassisted homebirths

Existential cultural factors or practices included preparations for delivery, seeking divine protection, daya’s practices during homebirths, and the use of dietary and herbal remedies. These practices had a neutral influence, neither positive nor negative, on decisions regarding seeking skilled birth attendants or unassisted homebirths. Participants described them as customary aspects of childbirth and culturally meaningful traditions. They were observed as birth care and healing practices and shaped the emotional and symbolic environment surrounding birth, aligning with the PEN-3 category of existential cultural influences. The following descriptions illustrate how childbirth occurred and was practically managed in home settings, in line with the objective of the study to document unassisted homebirth practices in Afghanistan.

### Existential perceptions

**Seeking Divine Protection:** The accounts showed that, recognizing the risks associated with childbirth, women sought divine protection and safety. As part of their preparation for childbirth, some women offered prayer in advance or during labour to seek protection from God. Additionally, some obtained a *Taweez* (amulet) from a mullah before labour began to ensure a smooth delivery. Taweez was believed to ward off any supernatural hindrances, such as the presence of *Peeryan* (ghosts), and to facilitate a trouble-free childbirth.

*I offered my prayer and prayed to be safe because death is likely during delivery. IDI Participant #7*

### Existential enablers

**Practices and Advice of Dayas in Assisting Homebirths:** Dayas provided hands-on care during homebirths using experiential and culturally normative techniques aimed at facilitating labour and delivery. Participants described how dayas assessed labour progression through observation and relied on positioning, physical pressure on the belly, and traditional remedies such as administering *Khak Sheer* to encourage contractions and assist delivery. Some participants described improvised techniques such as placing the woman on a rug held at the corners and shaken vigorously, which was believed to help push the baby down and speed delivery. Another method involved using a plastic sheet with two bricks, on which the woman was asked to sit and push in a squatting position. These practices reflected locally accepted knowledge systems rather than standardized protocols. For example, participant 7 described how the daya relied on improvised methods during her delivery. She recalled being positioned in various ways to encourage the baby’s arrival:

*She (daya) spread a plastic sheet on the floor and put two bricks on it. First, she asked me to push. When it was not helpful, then, she asked me to sit on the bricks. The baby was then born, after which she wrapped the baby and positioned me to lie on my side. IDI Participant #7*

Post-delivery practices included cutting and tying the umbilical cord using a blade or sometimes unconventional tools such as coins, often referred to as *“Panji”* [five Afghani coins], as a cutting instrument when a blade was unavailable. These accounts highlight how improvised cord-cutting methods reflected resource scarcity and raised concerns about the uncleanness of these practices. However, participants did not describe them as influencing decisions about birthplace or type of attendant.

*She cut the cord, tied it with thread, and put cotton on it. There were no gloves—she only washed her hands with soap. IDI participant #5*

In addition to dayas, family members were also typically present during childbirth. However, not all women preferred to have family members like their mother-in-law and sister-in-law present during their deliveries. Some women preferred having only a daya by their side, excluding family members. They found it embarrassing to have the family members present and allowed only the daya to assist during the delivery.

### Existential nurturers

**Preparation of Essentials for Mother and Baby’s Care:** During the pregnancy period, expectant mothers typically engaged in preparing essential items for the impending arrival of the newborn and supplies for the mother. Preparing essential items for the newborn typically involved sewing clothes, swaddles, and bedclothes—sometimes in special or decorative ways—to mark the birth as a significant occasion, with preparations often completed well in advance of the due date.

*I got a pair of clothes, fabric for a swaddle, and these things for the baby. This is a tradition in Afghanistan. IDI Participant #5*

Preparations also included procuring essential cleaning materials such as soap, shampoo, and a plastic sheet to ensure a clean place for both the mother and the newborn during delivery. Practical tools like thread and a blade for cutting the newborn’s umbilical cord were also prepared well in advance. However, not all deliveries occurred under sanitary conditions, and the umbilical cord was not adequately handled, particularly within low-income rural households. The family, particularly a female member, often the mother-in-law, took on the responsibility of ensuring all these essential materials were readily available on the day of the birth and conveniently stored near the birthing location.

*I bought some necessary things for the baby such as a blade, soap, and shampoo for bathing the baby. IDI Participant #6*

**Preparing the Physical Environment for Childbirth:** Home tidying was part of birth rituals, which was most pronounced in urban areas. Women engaged in thorough cleaning and organization of the home. Additionally, they undertook extensive laundry. Firstly, these activities were a tangible demonstration of the anticipation and welcoming of the newborn. Secondly, they provided a degree of assurance to expectant mothers. By ensuring that the home was clean and well-arranged, women effectively minimized the need to engage in strenuous activities during postpartum seclusion.

*When I knew it was the last month of my pregnancy and that childbirth was near, I arranged and cleaned the house, washed my children’s clothes, and completed all the other tasks. IDI Participant #09*

**Traditional Dietary Practices in Homebirths:** In advance of the birth, it was customary for expectant mothers or their family members to prepare and store items required for special diets for labour and postpartum. These diets can vary from region to region in Afghanistan and even from one household to another. Table 4 describes the common types and contents of traditional diets found by this study.

**Table 4:** Types of Diets.

Name of the diet	Contents	Time of intake
Milk or tea	Tea or boiled milk	During labour, taken warm
*Leeti* also called *Taasaw*	A soup consisting of flour, milk, sugar or jaggery (boiled and solidified sugarcane juice), and other additives, boiled in water	Right after childbirth
*Chaawa*	A watery mixture made from aniseed, jaggery, walnut, cardamom, and dried ginger, boiled in water	After childbirth for some days
*Roghan Zard*	Melted ghee (clarified butter)	Right after childbirth
*Yakhni*	Usually, chicken, water, spices/seasonings, e.g., black pepper, turmeric, and sometimes coriander seed	For several days after childbirth
*Roghan Donba*	Sheep tail fat meal, typically eggs fried in sheep tail fat.	After childbirth

During labour, women usually drank warm milk or tea, which was believed to alleviate discomfort, stimulate the labour process, and aid in a smooth delivery. Women reported that they take it when the labour pain starts. Sheep tail fat was highly regarded for its perceived nutritional benefits for postpartum women. Eggs fried in sheep tail fat was a common postpartum meal. Additionally, traditional porridges, such as leeti, taasaw, and chaawa, were believed to promote the well-being of both the mother and the child during the postpartum period. These traditional foods had the dual purpose of aiding in milk production for the mother and nourishing her. Yakhni was often given to women after childbirth as it was believed to have restorative and nourishing properties for women during postpartum. In certain communities, immediately after childbirth, the mother-in-law prepared ghee [purified butter] or special oil for the mother to consume, with the intention of helping her regain her strength.

Participants 7 and 8 explained the traditional foods they consumed after delivery to help restore the mother’s strength and promote milk production:

*I made chaawa. These things are given to the mother to keep her warm and produce milk for the baby. IDI Participant #7*

*I had leeti or taasaw, milk, and eggs. I could not stand up for three days. One side of my body had colic pain. I was so weak that others had to feed me taasaw with a spoon. IDI Participant #08.*

The dietary choices of pregnant women were often dictated by family members, especially the mother-in-law and other senior women, a practice most pronounced in extended families. The daughter-in-law was expected to comply with these dietary instructions, highlighting the limited autonomy of women in this regard. For instance, a participant said that her mother-in-law, influenced by her own experiences and cultural beliefs, discouraged the consumption of milk during pregnancy due to a perceived risk of miscarriage. Even though she wanted to drink milk during her pregnancy, the woman had no autonomy to refuse her mother-in-law’s instructions. Another woman shared that right after giving birth at home, a meal made from sheep tail fat was prepared for her. She had to eat it despite finding it difficult to consume it due to its unpleasant smell that made her nauseous. This was because her family members insisted that she should eat it, and she had no choice but to comply.

**Remedies and Healing Traditions in Homebirths**: Participants’ accounts indicated that families adopted various practices to ensure women’s well-being after giving birth at home. Table 5 shows a summary of the practices and remedies:

**Table 5:** Types of Medicines, Remedies, and Healing Practices in Homebirths.

Remedy	Preparation	Time of use
*Khak Sheer* mixture	Flixweed seed boiled in water	Right after childbirth
Dough therapy	Dough wrapped around the head	After childbirth
Belly binding	Securing a cloth around the belly	A few days after birth
Seclusion	Resting and avoiding heavy tasks	After birth, for some days or weeks.

As a protective measure, some women wrapped a cloth around their bellies, providing stability and support to the abdominal area. This practice was rooted in the belief that it helps in the recovery of abdominal muscles and minimizes discomfort during the postpartum period. In certain communities, immediately after childbirth, the mother-in-law wrapped dough around the woman’s head, with the intention of helping her regain her strength. Some of them ended up receiving *Unani* [Greek] medicines like khak sheer to address postpartum bleeding.

*After I gave birth, the daya prepared khak sheer and gave it to me to drink to stop the bleeding. IDI Participant #7*

One common postpartum practice was the observance of a period of postpartum seclusion during which the mother and child stay indoors. The period typically lasted for 4–5 days and sometimes extended to a few weeks. This period served as a crucial time for rest and recovery. During seclusion, women exercised caution in their daily activities, often choosing to abstain from heavy tasks. Throughout the seclusion period, a family member, usually the mother, sisters, or a relative from either the woman’s or her husband’s family, took on the role of caregiver. This designated caregiver not only looked after the postpartum woman but also assumed responsibility for household chores. This arrangement allowed the new mother to focus on her recovery and bonding with her newborn, relieving her of the burden of domestic responsibilities. Once the seclusion period concluded, women gradually resumed their daily activities, starting with lighter tasks and progressively moving towards heavier ones.

*After delivery, I rested at home for 4 or 7 days, and then I started doing chores. IDI Participant #8*

### Theme 3: Negative cultural influences on unassisted homebirth

This section focuses on core cultural norms and authority structures shaping unassisted homebirth decisions, illustrated through selected representative accounts. These negative cultural influences hindered women’s access to skilled birth attendants and reinforced reliance on unassisted homebirths.

### Negative perceptions

**Perception of Facility Birth as *Sharm* [disgrace]:** Participants mentioned that in some communities, especially in rural areas, delivery in a health clinic or hospital was not culturally acceptable because of the perception that doctors touch female bodies and women’s fear of being seen by strange men during childbirth. Hence, participants indicated that seeking medical care for childbirth was regarded as *Sharm* [a disgrace] in their communities, or some families believed that giving birth in a hospital with a male presence carries a social stigma.

For instance, participant 4 further explained how daughters-in-law were discouraged from facility births because of strong social norms around modesty and gender segregation. She noted that family members considered it disgraceful for women to be examined by doctors, seen by male clients in the health care facility, or even observed by a male gatekeeper at the hospital:

*They (family members) tell them (daughters-in-law) that it is a big sharm that a doctor-sorry for frankly saying it- touches you by hand. There are male clients in the clinic, there is a Baba (male gatekeeper) in the hospital. It is sharm that a man (male gatekeeper) sees you with this big belly. That’s why they [daughters-in-law] did not go to the hospital. IDI Participant #4*

**Reliance on Faith and Destiny:** A strong reliance on faith and destiny over medical intervention, especially encouraged by family elders, was a barrier to seeking skilled birth care. Participants recounted seeking her mother-in-law’s and father-in-law’s help to accompany her to the hospital for childbirth. However, they encouraged her to deliver at home, reassuring her with their belief in God’s kindness and the idea that divine actions surpass human efforts. Participant 8 recounted experiencing labor alone and placing her trust entirely in God’s will. She explained that prayer was her primary response to the uncertainty of childbirth:

*I was in labour and alone. I just trusted God and thought if the child’s destiny was to come to the world, it would happen. I offered two Rakat [units] of prayer. IDI Participant #8.*

**Facility Delivery Considered Only for Complications**: The accounts indicated that delivery was often regarded as a natural event that did require medical assistance. In specific communities, especially in rural areas, the primary motivation for families to bring the expectant mother to a healthcare facility for delivery was the potential complications during labour. Otherwise, unassisted homebirth remained their initial choice. For instance, participant 4, a woman from a rural community, explained that although her first child was born in a hospital, this decision was only made because of complications that required medical attention. She reflected that, without those complications, she would also have delivered her first child at home:

*If I hadn’t faced birth complications and didn’t need an operation, I would have delivered my first child at home as well. IDI Participant #4*

**Trust in Dayas as Knowledgeable Birth Attendants:** Dayas were traditionally seen as knowledgeable and experienced individuals, often readily available in the community to assist with childbirth. Participants emphasized that dayas did not conduct vaginal examinations, which was often seen as a more respectful approach compared to doctors or other skilled birth attendants. Dayas were often selected based on their reputation for successfully assisting with deliveries in the community, and are frequently recommended by family members, especially mothers-in-law who believed in their capabilities and expertise. In some cases, they had a record of assisting with multiple generations of childbirth within a family. For example, participant 5 explained how her mother-in-law insisted on calling the same daya who had delivered several children in their family, emphasizing her perceived wisdom and skill:

*My mother-in-law said daya should come because she delivered everybody in our family. She should also deliver you, as she is a knowledgeable and wise woman, and she knows what to do. IDI Participant #5*

Similarly, participant 4 contrasted the practices of dayas and doctors, highlighting that dayas avoided frequent vaginal examinations, which she and other women in her community viewed as more respectful than the approach of skilled providers:

*Daya never examines vaginally. She waits for the woman to deliver. She waits, and when the child is born, she tells you that your baby was born. However, the doctor examines you every while, frequently, vaginally. It is sharm for women. IDI Participant #04.*

### Negative enablers

**Reliance on Relatives and Dayas for Birth Care:** The data suggest that most families in rural areas rely on dayas or relatives as the first option for birth assistance. When labour began, daughters-in-law typically sought guidance from their mothers-in-law or other experienced women in the family, whose advice was routinely sought and formed an integral part of cultural and traditional practices. The family typically guessed the progress of labour by speculating on the intensity of the pain. As labour pain intensified, families first sought a traditional birth attendant to aid in the delivery. Participants cited a range of homebirth attendants. The attendant was informed some days ahead of the estimated week or days of delivery or called by a family member when the mother was in labour. Most participants recounted that daya played a central role in assisting with homebirths in communities.

*My father-in-law called his uncle’s wife, who was a daya and helped with the last 3–4 deliveries of my mother-in-law, to help me as well. IDI Participant # 4*

Some participants living in urban areas also reported seeking assistance from trusted and experienced family members such as mothers in law and experienced female relatives, as well as neighbors or senior women within their community. Accounts show, particularly in cases of rapid labour development, that family members often assumed the role of birth attendants themselves, especially when there was no time to seek external help. Participant 9 explained that she relied on her mother-in-law for practical tasks during labour and after birth, including caring for her, assisting with the newborn, and cutting the umbilical cord:

*My mother-in-law assisted with my birth. Thanks to God; my child did not move for some while. My mother-in-law played the role of a doctor. She shook the baby, pressed the baby’s chest, put a finger in the child’s mouth, and pressed its nose. The child cried. IDI Participant #8.*

### Negative nurtures

**Homebirth Upheld as a Traditional Norm [*Rawaj*]:** Most participants mentioned that homebirth is seen as a tradition, also called *Rawaj*, passed on from one generation to another, and a common practice in their communities. Embracing homebirths exclusively has evolved into a familial legacy, compelling women to adhere to this practice regardless of the challenges they may encounter. Participant 5 described how the practice of homebirth was embedded in family tradition, noting that some women in her community had delivered all of their children at home without ever seeking hospital care:

*It is their [their family] rawaj (tradition), and that’s why all of them (women) give birth at home. There were some women (in our community) who gave birth to 10 children at home, just at home. They have never gone to a hospital. IDI Participant # 5*

Accounts show that mothers-in-law often insisted on unassisted homebirths as rawaj, a family and community tradition, and discouraged the involvement of medical assistance. The advice of the mothers-in-law was highly regarded, and their opinions carried considerable weight. Together with the husband and father-in-law, mothers in law were influential family elders. This strong stance stemmed from the mother-in-law’s beliefs surrounding childbirth, where she asserted her own and her relatives’ experiences of giving birth at home without any modern maternal care as a basis for guiding her daughter-in-law’s choices. Beyond individual authority, accounts also show that the continuation of homebirth within some extended families fostered a sense of familiarity and comfort with the practice. Participant 7 described how her sister’s mother-in-law insisted on maintaining the tradition of homebirth, pointing out that nearly all children in the family had been delivered at home rather than in a hospital:

*Her (my sister’s) mother-in-law said, “This is not common in our tribe; she should give birth at home.” Only two children in their family were born in a hospital, while the rest were born at home. IDI Participant #7*

**Deference to Elders’ Decisions in Childbirth Care:** Accounts show that it was a commonly accepted family norm that the parents are the elders of the household and possess wisdom and experience that should not be challenged. As a result, married sons, especially in the rural villages, often did not feel entitled to voice their opinions against the decisions made by their father and mother, including those related to the choice of homebirth. In many cases, husbands followed the decisions of their parents, leading to homebirth even when their wives preferred hospital delivery. Junior women, especially daughters-in-law, had limited autonomy when it came to deciding where to give birth.

*My mother-in-law decided that I should give birth at home. There are families in my community that even do not let women go to the doctor during their pregnancy. They say we have never taken a pill during our pregnancies, so why should you take it? They do not let them go to the doctor. The daughters-in-law cannot say anything (cannot oppose). IDI Participant #3*

**Veiling and seclusion practices as barriers to facility-based childbirth:** Accounts from women’s FGD show that in some communities, when a woman married and joined her husband’s family, she was prohibited from being seen by her brothers-in-law and father-in-law, a restriction that remained in place until she had given birth to several children. To maintain this tradition, the woman wore a veil or shawl, concealing her face and ensuring that she remained hidden from view and secluded at home for several years after marriage. Consequently, her childbirth took place at home, either alone or with the assistance of an elderly female family member.

*In some communities, when a woman gets married and joins her husband’s family, her brothers-in-law and father-in-law are not allowed to see her, and this restriction can continue until she has several children. The bride always wears a veil or shawl to cover her face and stays secluded in her room. This is a tradition in some rural communities. Women’s FGD Participant #4*

**Circumstances Resulting from Delayed *Toyana or Qaleen* [dowry]:** Based on the narratives from FGDs, there were unique cases where women with pregnancies out of wedlock secretly delivered their children at home. This situation typically arose when women’s fiancés encountered difficulties in fulfilling the customary obligation of providing the *Toyana* or *Qaleen*, a monetary payment to the bride’s family. As a result, their weddings were postponed, and the women continued to reside at their parents’ houses. In some cases, couples had already entered into a formal marriage agreement, known as *Nikah* [an Islamic contract that legally and religiously binds a man and a woman as husband and wife], while in others, no such agreement had yet been made. During this period, the women and their fiancés maintained a discreet relationship. These women concealed their pregnancies to avoid stigma, giving birth discreetly at home, either in secrecy or with the support of a daya. This secretive childbirth often resulted in the child being labeled *Najayez* [illegitimate—children born out of wedlock], symbolizing its unauthorized status within cultural or religious contexts.

*They became pregnant before marriage to their fiancé, prior to the wedding party, which is a one-day celebration usually held at the bride’s father’s home. Although a Nikah had been performed, the child born to them is considered najayez in Afghanistan. Most najayez births take place at home without anyone’s assistance. Women’s FGD Participant #4*

**Childbirth in Solitude:** In certain instances, women endured labour and childbirth in solitude, without the assistance of a daya or any family members. This isolation often arose from inadequate birth preparation or family negligence, leading to the absence of support during labour. These women navigated childbirth independently, relying solely on their instincts and faith. In such circumstances, women took on the responsibilities of cutting the umbilical cord and swaddling the newborn immediately after birth themselves. Participant 8 described giving birth entirely alone at night when no one was available to help her. Despite calling for assistance, cultural restrictions and family dynamics left her unattended until after the birth:

*In my other delivery at home, nobody was there, I helped myself. It was nighttime. My labour pain started around ten o’clock in the evening. I was walking and screaming in the yard until the baby was born at about four o’clock in the morning. My children were all asleep. I called our neighbor and asked her to come and help with my delivery, but her husband did not let her come and help me. Then, the baby was born. My sister-in-law and other relatives came in the morning and saw me with my baby swaddled and sleeping next to me. IDI participant #08*

## Discussion

Our study examined unassisted homebirths in Afghanistan to identify the cultural factors influencing this practice, categorizing them as positive, existential (neutral), and negative, guided by the PEN-3 model [31]. It further aimed to document the practices surrounding preparations for childbirth, labor and delivery, and the postpartum period within the Afghan cultural context. While our findings indicate that cultural practices contribute to decisions about unassisted homebirth, these influences must be understood within the broader structural and political context, including insecurity, limited health system capacity, and restrictions on women’s access to care under Taliban rule.

These findings indicate that awareness of childbirth risks and male support, particularly from educated husbands, can partially counterbalance entrenched family hierarchies, especially in nuclear households. Similarly, a scoping review of skilled birth attendance utilization in low- and middle-income countries found that concerns about birth complications and the influence of an educated husband positively contributed to the use of skilled birth attendance [48]. This finding highlights the potential of male engagement and education in improving maternal care decisions.

The existential practices identified in this study highlight how childbirth is embedded in culturally normative routines that shape women’s lived experiences of labour without necessarily determining care-seeking behaviour. These practices in the Afghan families function as stabilizing elements that provide familiarity, comfort, and perceived safety during childbirth, even in the absence of biomedical care. Their persistence suggests that efforts to promote skilled birth attendance must account for these culturally embedded practices rather than attempt to replace them outright. Qualitative studies in other South Asian countries have reported similar practices. In Karachi, Pakistan, women engaged in religious rituals during homebirths, such as offering prayers for divine protection [49]. Women in India and Nepal consumed traditional postpartum foods—*Sooth-gur Ladoo* in India [made with ginger, jaggery, dry fruits, coconut, and spices] and *Kwati* in Nepal [a bean soup served with meat, curd, beaten rice, and meat curry]—to support recovery after childbirth [50,51]. Postpartum seclusion was also documented in Pakistan, Nepal, and Sierra Leone as a cultural norm to promote rest and recovery following childbirth [50,52,53]. These practices, rooted in cultural identity in many low-income countries, do not explicitly promote or hinder the utilization of skilled care.

The negative cultural influences identified in this study reflect entrenched authority structures and moral expectations that regulate women’s reproductive behaviour. Practices such as deference to elders, framing facility birth as sharm, and reliance on dayas are not isolated beliefs but interconnected norms that collectively limit women’s autonomy in childbirth decision-making. These findings demonstrate how cultural authority is exercised within families to maintain continuity of homebirth practices, even when women themselves recognize potential risks. Together, these findings suggest that unassisted homebirth in Afghanistan is sustained less by individual preference than by collective enforcement of cultural norms embedded in family hierarchies and gendered expectations.

While this study centers on cultural drivers of unassisted homebirths, it is vital to recognize how these practices and beliefs intersect with broader structural issues, such as insecurity, inadequate health infrastructure, and geographic remoteness, that significantly restrict access to facility-based maternal care in Afghanistan. For example, insecurity, geopolitical instability, interrupted health system funding, and escalating disparities in maternal and child health coverage, especially in remote and socioeconomically disadvantaged regions, have intensified inequities across the country [54,55]. Additionally, the results of this study underscore that unassisted homebirth in Afghanistan is not only a matter of personal choice or cultural tradition but is also shaped by the governance under the Taliban rule. Recent qualitative evidence from Afghanistan under the Taliban shows that bans on women’s education, work and movement have reduced the availability of female healthcare providers and constrained access to formal health care services, including health information, which often leads women to rely on informal or traditional channels like *mullahs* for maternal care [54]. These intersecting cultural and structural constraints underscore the need for approaches that address both sets of determinants to improve maternal health outcomes.

A key contribution of this study is its in-depth examination of how cultural norms, family authority, and childbirth practices influence decisions around unassisted homebirth in Afghanistan. By situating these cultural beliefs and practices within the contemporary socio-political and health system context, our findings show that culture is not a static set of traditions but is shaped and reinforced under conditions of insecurity, governance constraints, and limited access to skilled care. This perspective helps explain why unassisted homebirths persist even where physical access to facilities exists and underscores the importance of addressing cultural beliefs and practices alongside broader structural realities.

### Limitations

The study has a number of limitations that should be acknowledged. First, the data relied on narratives from Afghan refugee women residing in Canada. Migration may have influenced how participants remembered and reflected on their childbirth experiences. While these participants provided rich accounts of unassisted homebirths in Afghanistan, refugee participants may constitute a more selective group, and their narratives may not capture the full spectrum of childbirth experiences across Afghanistan’s diverse sociocultural landscapes. Second, IDI participants reflected on birth experiences that occurred three to nine years prior to data collection, which may have influenced the level of detail in some narratives due to possible recall limitations. Third, variations in educational attainment across participants may have shaped the range of perspectives shared. While one-third of IDI participants were illiterate, most FGD participants had university-level education, which could have influenced how experiences were articulated and the emphasis placed on particular aspects of childbirth practices. Despite these limitations, this study contributes original qualitative evidence on unassisted homebirths in Afghanistan, offering insights into an underexplored area and providing a foundation for future research.

## Conclusion

This study provides a detailed cultural account of childbirth in the context of Afghanistan. The findings suggest that efforts to improve skilled birth attendance should work through, rather than bypass existing family authority structures and culturally embedded birth practices. Participants consistently described dayas as trusted figures whose authority derives from experience, accessibility, and social acceptance rather than formal training. Any engagement with dayas should therefore recognize their role as cultural brokers within communities, rather than positioning them as replacements for skilled providers.

The findings contribute to ongoing discussions about the potential role of dayas within maternal healthcare strategies. Carefully defined engagement with dayas should focus on supportive, non-clinical roles rather than medical care. In this role, dayas could provide emotional support during labour, help families communicate and coordinate with community midwives, and encourage timely referral to health facilities when complications arise. Alongside this, strengthening community midwifery through culturally informed training and limited incentives for home-based skilled care may support gradual transitions from unassisted homebirths toward safer birth practices. While community midwives represent a culturally acceptable form of skilled care, their deployment remains constrained under current conditions due to restrictions on women’s education, mobility, and employment, which limit the scalability of midwifery-led interventions.

Any efforts to expand community-based maternal health services would therefore require careful negotiation with de facto authorities, the Taliban. Under current conditions, there is limited but meaningful acceptance of women-to-women healthcare provision, creating a narrow operational space for female-delivered maternal care. Such engagement must remain attentive to ethical concerns, women’s autonomy, and the risk of reinforcing restrictive gender norms. Taken together, these findings suggest that incremental, culturally grounded approaches, rather than rapid system-level reforms, may represent a feasible pathway for improving maternal safety in Afghanistan’s current sociopolitical context.
